# A genomic strategy for precision medicine in rare diseases: integrating customized algorithms into clinical practice

**DOI:** 10.1186/s12967-025-06069-2

**Published:** 2025-01-20

**Authors:** Cristina Méndez-Vidal, Nereida Bravo-Gil, Javier Pérez-Florido, Irene Marcos-Luque, Raquel M. Fernández, José Luis Fernández-Rueda, María González-del Pozo, Marta Martín-Sánchez, Elena Fernández-Suárez, Marcela Mena, Rosario Carmona, Joaquín Dopazo, Salud Borrego, Guillermo Antiñolo

**Affiliations:** 1 Institute of Biomedicine of Seville, IBiS/University Hospital Virgen del Rocio, CSIC/University of Seville, Seville, Spain; 2 Centre for Biomedical Network Research on Rare Diseases (CIBERER), Seville, Spain; 3 Platform of Computational Medicine. Fundación Progreso y Salud (FPS). CDCA, University Hospital Virgen del Rocio, Seville, Spain; 4 Department of Maternofetal Medicine, Genetics and Reproduction, Institute of Biomedicine of Seville, IBiS/University Hospital Virgen del Rocio, CSIC/University of Seville, Seville, Spain

**Keywords:** Rare diseases, Genetic diagnosis, Next generation sequencing, Precision medicine, Research implementation, Genomic medicine

## Abstract

**Background:**

Despite the use of Next-Generation Sequencing (NGS) as the gold standard for the diagnosis of rare diseases, its clinical implementation has been challenging, limiting the cost-effectiveness of NGS and the understanding, control and safety essential for decision-making in clinical applications. Here, we describe a personalized NGS-based strategy integrating precision medicine into a public healthcare system and its implementation in the routine diagnosis process during a five-year pilot program.

**Methods:**

Our approach involved customized probe designs, the generation of virtual panels and the development of a personalized medicine module (PMM) for variant prioritization. This strategy was applied to 6500 individuals including 6267 index patients and 233 NGS-based carrier screenings.

**Results:**

Causative variants were identified in 2061 index patients (average 32.9%, ranging from 12 to 62% by condition). Also, 131 autosomal-recessive cases could be partially genetically diagnosed. These results led to over 5000 additional studies including carrier, prenatal and preimplantational tests or pharmacological and gene therapy treatments.

**Conclusion:**

This strategy has shown promising improvements in the diagnostic rate, facilitating timely diagnosis and gradually expanding our services portfolio for rare diseases. The steps taken towards the integration of clinical and genomic data are opening new possibilities for conducting both retrospective and prospective healthcare studies. Overall, this study represents a major milestone in the ongoing efforts to improve our understanding and clinical management of rare diseases, a crucial area of medical research and care.

**Supplementary Information:**

The online version contains supplementary material available at 10.1186/s12967-025-06069-2.

## Background

Given that 80% of rare diseases have a genetic component, delivering genomic information in a timely manner has proven to enhance clinical decision-making, improve patient outcomes, and facilitate personalized treatment strategies, ultimately leading to more effective management of complex diseases [1]. In this light, Next-Generation Sequencing (NGS) has emerged as the gold standard for the genetic diagnosis of most hereditary disorders. Due to its analytical accuracy, high throughput, and cost-effectiveness, NGS is playing a crucial role to provide a molecular diagnosis, particularly for patients with inherited rare diseases or familial cancer [2], shaping the implementation of precision medicine in public health systems [3].

Despite the efforts and advances to integrate NGS into clinical practice, various factors continue to hinder its effective implementation, causing delays in timely diagnosis [4]. These challenges include the lack of standardized methods for interpreting complex genomic data, leading to variability that complicates clinical decision-making. Furthermore, issues related to quality assurance, along with persistent concerns regarding cost and reimbursement, add additional complexity to the integration process. Addressing these factors is crucial for the effective use of NGS in molecular diagnosis. However, given the disparity of factors, a multifaceted approach is needed for a successful implementation [5]. The current study has addressed manageable aspects with immediate applicability to NGS adoption, offering a starting framework for other mid-sized laboratories. These include optimization and standardization of laboratory protocols, definition of virtual panels, improvement of interdepartmental communications, establishment of data handling solutions, and development of a corporate bioinformatics tool for NGS data analysis and semi-automated reporting.

While a growing body of evidence highlights the benefits of genome and whole exome sequencing in clinical practice [2], multi-gene panel sequencing still remains as a reasonable initial diagnostic step for many laboratories, due to its cost-effectiveness, faster turnaround time, reduced risk of incidental findings and uncertain variants, lower storage needs, and greater flexibility in allowing the deliberate capture of non-coding regions. Additionally, it provides higher coverage, limiting the need for ad hoc additional analysis compared to other large-scale NGS approaches [6, 7]. This has been of particular interest in areas such as oncology and single-system diseases [8].

Various commercial solutions are available to assist clinicians and geneticists in different points of the diagnostic process, including probes design, genomic data analysis or variant interpretation [9]. However, maintaining a high degree of understanding and control over the whole diagnostic process is crucial in the clinical setting. Generally, this is achievable when using tailored tools including custom designs, flexible and appropriate virtual panels and in-house data analysis pipelines [10].

Digitization of healthcare is generating unprecedented amounts of data which can lead to disruption, duplication and underuse [11]. This situation is evolving towards global analysis models, allowing the connection of records and the interoperability of information systems [12]. In fact, an integrated care is particularly relevant to the genetic diagnosis of patients with complex multisystem disorders [13], which encompasses a large proportion of rare genetic diseases, as it allows simultaneous genetic analysis using the same sequencing data. Remarkably, corporate bioinformatics solutions integrated into the medical record would enhance data privacy by avoiding the challenges related to sharing patient data on commercial clouds [14]. These advantages would be reinforced by integrating them into a health system that is connected to a distinctive centralized medical records model, ensuring equality in the provision of services, non-fragmented healthcare, and the accessibility to clinical findings. Considering all these features, the Andalusian healthcare system constitutes an excellent model for the integration of clinical and genomic data since: (i) it covers the largest autonomous community in Spain with a population of nearly 9 million inhabitants, (ii) it has a unified digital clinical record interoperable and accessible from any public health center in the Andalusian territory and (iii) it already has experience in successful projects aimed to overcome the difficulties associated with managing genomic data [15–17].

Undoubtedly, NGS is a powerful tool that has revolutionized the field of genomics and molecular biology. By enabling comprehensive analyses at a genomic scale, NGS aids in the development of targeted therapies, and enhances our understanding of complex diseases. Our approach, in conjunction with a universal healthcare system, has improved the efficiency of genetic diagnosis to streamline the implementation of NGS in the Andalusian public healthcare system as we have taken the necessary steps to move towards the integration of genomic and clinical data.

## Methods

### Study participants

**Fig. 1:**
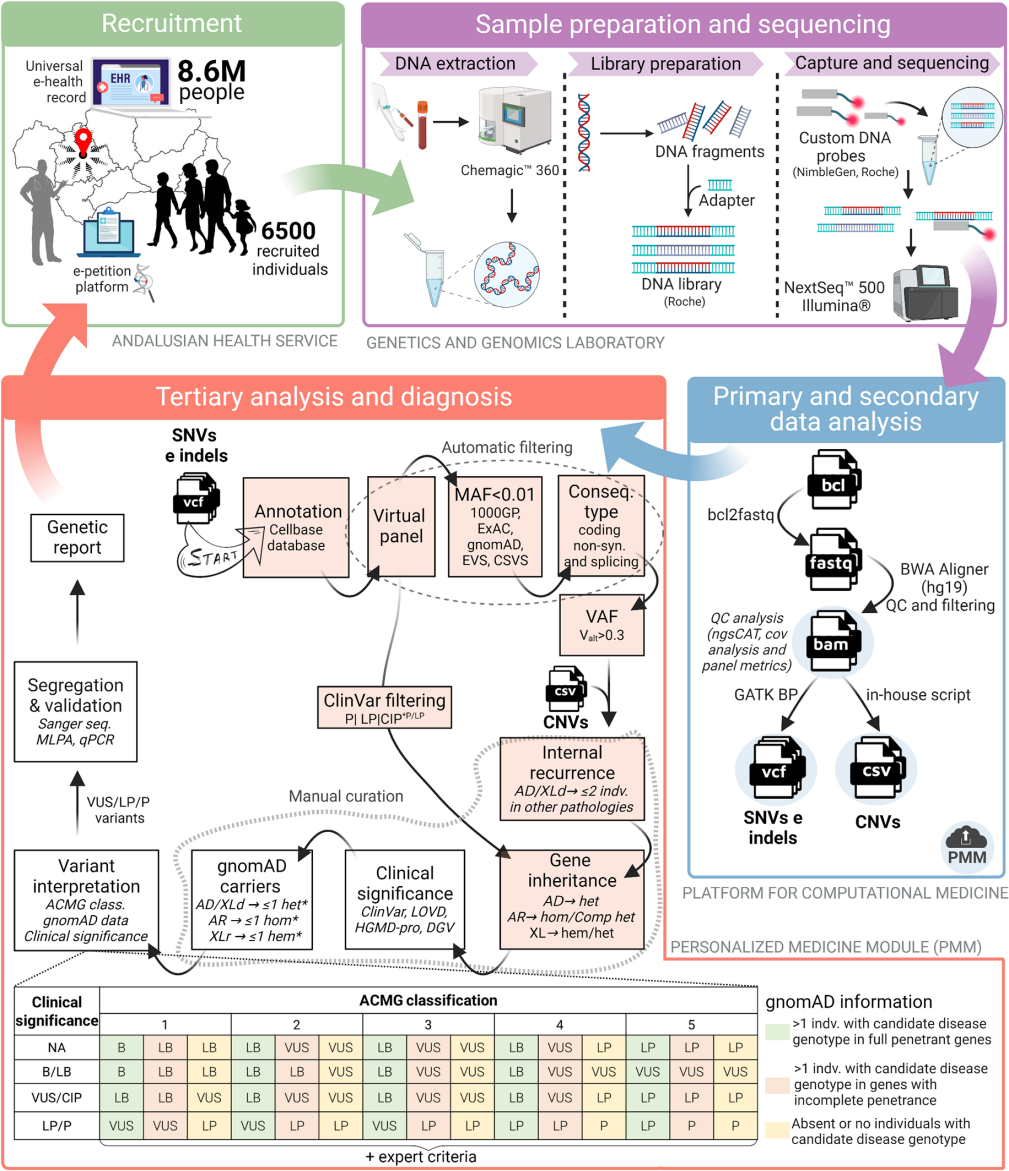
Overview of an NGS-based pilot program for genetic diagnosis of rare diseases in a public reference healthcare hospital. All recruited individuals came from one of the eight provinces of Andalusia and were included into the study according to their clinical manifestations or family history, through an electronic platform filled out by the clinician responsible for the genetic study request. After library preparation, sequencing and the automatic phase of data analysis, the alignment, quality control (QC) and variant files were uploaded to the personalized medicine module (PMM) for evaluation by the geneticist. Once the variant interpretation was finished, the geneticist could prepare a genetic report accessible to patients and their requesting clinician in the electronic health record. *Except for genes with variants previously reported as pathogenic, hypomorphic alleles and variants showing incomplete penetrance or variable expressivity. Abbreviations: ACMG class, American College of Medical Genetics and Genomics and Association for Molecular pathology classification; AD, autosomal dominant; AR, autosomal recessive; B, benign; CIP, conflicting interpretations of pathogenicity; CNVs, Copy Number Variations; Comp het, compound heterozygous; Cov, Coverage; GATK BP, Genome Analysis ToolKit Best Practices; Hem, hemizygous; Het, heterozygous; Hom, homozygous; Indv, individual ; LB, likely benign; LP, likely pathogenic; MAF, minor allele frequency; P, pathogenic; SNVs, single nucleotide variants; VAF, variant allele fraction; VUS, variant of uncertain significance; XL, X-linked; XLd, X-linked dominant; XLr, X-linked recessive. Created with BioRender.com

This study is a retrospective review of 6500 consecutive NGS tests (6267 diagnostic assays and 233 carrier studies) performed at the Department of Maternofetal Medicine, Genetics and Reproduction, of University Hospital Virgen del Rocio (Seville, Spain) from January 2018 to December 2022. An overview of this NGS-based pilot program is shown in Fig. 1. Subjects included in the study were referred to our Department for genetic testing using a corporative application for electronic prescription of laboratory tests. Individuals referred for evaluation of carrier status or presymptomatic testing were excluded from the NGS data analysis, except for preconception, condition-directed studies in carrier couples at increased risk of having affected offspring, as well as for the screening of technically challenging variants associated with the use of Sanger, MLPA or qPCR.

The genomic DNA (gDNA) extraction and quality assessment was performed as described in Additional file 1. Collected samples were stored as part of a DNA collection, which was registered in the National Biobank Registry of the Institute of Health Carlos III with the reference number C.0004010.

### Design and development of a personalized Rare diseases Exome (pRARE)

To implement routine genetic testing for all inherited disorders included in the services portfolios of all public Andalusian hospitals, we designed the first version (D1) of an NGS custom designed panel called pRARE (Personalized Rare Diseases Exome), which was subsequently updated twice (D2 and D3, consecutively) with the purpose of improving coverage of the most in-demand groups of pathologies. These designs (see Additional file 2 for further details) included all exons (D1) or coding exons (D2 and D3), exon-intron boundaries and selected deep-intronic regions for 886 genes (D1), 1163 genes (D2) or 1397 genes (D3), globally covering more than 1800 rare inherited conditions. The details of probes design and statistical methods used to compare the diagnostic yield of each version per disease category are described in Additional file 1.

### Establishment of virtual panels

Diagnostic-grade virtual panels were defined based upon a review of medical literature and public databases. Moreover, we also collected clinical and scientific information from different disease area experts to reach a consensus on which genes had sufficient evidence for disease association and to gather any additional genes that should be included. Upon clinical evaluation of the patient, referring clinicians or geneticists had the flexibility to choose from these pre-defined panels, customize these panels with additional genes, or order multiple panels for a single patient.

### Library preparation, sequencing and bioinformatic analysis

One microgram of gDNA was used for library preparation (Fig. 1) using the SeqCap EZ Library SR version 5.1 for D1 and D2, or KAPA Hyperplus Kit version 3.1 for D3 (Roche, Indianapolis, IN, USA) according to the manufacturer’s protocol with minor modifications (see Additional file 1 for further details). Sequencing was performed in the Illumina NextSeq500 platform (Illumina, USA). Following sequencing, alignment and variant calling were performed using a previously validated single informatics pipeline for multiple rare genetic tests [18], with some modifications (see Additional file 1). The result of the application of this pipeline was a VCF file for each sequenced sample. In parallel, an in-house independent script was designed and implemented for the analysis of copy number variations (CNVs) based on coverage and statistical studies.

### The Personalized Medicine Module

**Fig. 2:**
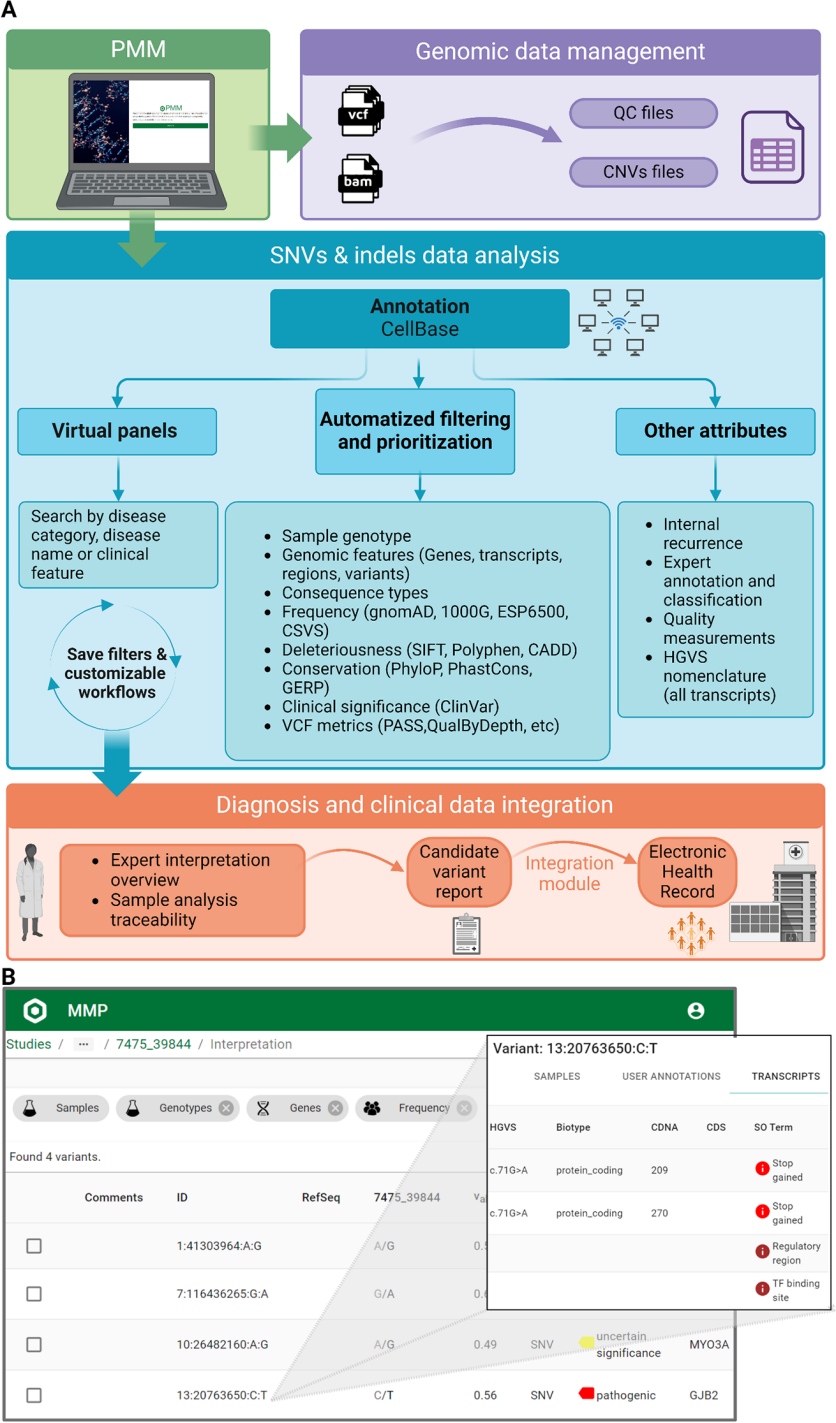
Overview of the personalized medicine module (PPM) tool. **(A)** The PMM tool incorporates functionalities for genomic data management, SNV/Indels analysis and integration into the clinic. After primary and secondary data analysis, VCF, BAM and BAI files are uploaded into the PMM data analysis module, as well as sample QC and CNVs files. Once the study sample is selected, users can automatically apply both virtual panels and a set of filters, together with other complementary attributes to reach a manageable number of diagnostic variants. The custom combination of filters and prioritization settings can be saved for further analyses, as well as the variant-associated annotations and expert classifications to facilitate variant interpretation. Finally, the tool has the possibility to semi-automatically generate a variant report to be integrated into the patient’s electronic health record. **(B)** PMM tool screenshots showing the prioritization module interface and the detailed variant window. Created with https://BioRender.com

The Personalized Medicine Module (PMM) is an evolved version of a bioinformatic tool for variant prioritization [19], with a strong focus on clinical utility [20]. Briefly, it is a web-based interface with a backend that indexes VCF files and annotates them using a locally enhanced version of CellBase [21]. The frontend of PMM allows users to query the annotated data and prioritize the most likely causal variants. Variant prioritization was carried out based on different criteria such as clinical databases classification [22], distinct pathogenicity indexes (e.g. Polyphen [23], SIFT [24], etc.), population frequencies, human phenotype ontology [25], virtual panels, etc. Figure 2 depicts the general operation of PMM.

### Validation of the NGS-based approach

To ensure a consistent high standard of performance, our approach was externally validated by the participation in the EMQN External Quality Assessment Scheme for NGS-based germline variants testing. Additionally, the PMM tool was validated and optimized, both using a control set of 139 pseudocontrols samples harboring 163 known genetic variants (Additional file 3), and a double analysis in 1488 samples with unmet diagnosis, using the open-source application wAnnovar [26], as previously described [27]. On the other hand, potentially pathogenic variants with sequencing depth below 20x were confirmed by Sanger sequencing according to the manufacturer’s protocols (3730 DNA Analyzer, Applied Biosystems, Foster City, CA, USA). In addition, CNVs were confirmed by Multiplex ligation-dependent probe amplification (MLPA, MRC-Holland, Amsterdam, The Netherlands), using a 3730 Genetic Analyzer (Applied Biosystems) and the GeneMarker software. Alternatively, quantitative PCR (qPCR) was performed using SsoAdvanced™ Universal SYBR^®^ Green Supermix (BioRad) and the Applied Biosystems ABI 7500 thermocycler. The calculation of the fold change of CNVs was performed by using the comparative Ct (2^−ΔΔCt^) method and negative control samples as references.

### Tertiary analysis of identified variants

The first step in the tertiary analysis was the selection of the virtual panel according to the clinical diagnosis given by the referring clinician. A structured workflow for prioritizing SNVs and small indels using the PMM tool was developed (Fig. 1). Variants were prioritized based on established clinical and genetic criteria (see Additional file 1 for a complete description of the workflow). Likewise, for the prioritization of CNVs, we defined gene dose and z-score parameters during the CNVs validation, using 26 patients harboring known CNVs (Additional files 1 and 3).

### Interpretation and clinical reporting of variants

Semi-automated variant interpretation was done using Franklin (https://franklin.genoox.com/clinical-db/home) and/or Varsome (https://varsome.com/). This classification was completed with laboratory data and expert criteria to reach the final categorization, always following the recommendations of the American College of Medical Genetics and Genomics (ACMG) and the Association for Molecular pathology (AMP) [28]. Only pathogenic/likely pathogenic variants or variants of unknown significance compatible with the phenotype, were further considered for segregation studies (Fig. 1) using Sanger sequencing or MLPA/qPCR, discarding those not consistent with the pedigree. Remaining variants were reported to referring clinicians and patients. When no causative variants were identified, a negative report was emitted. The nomenclature of variants was adjusted to the Human Genome Variation Society version 21.0.2 (https://hgvs-nomenclature.org/stable/) guidelines using Mutalyzer version 3.0.6 (https://mutalyzer.nl/).

## Results

### Clinical description of the subjects

**Fig. 3:**
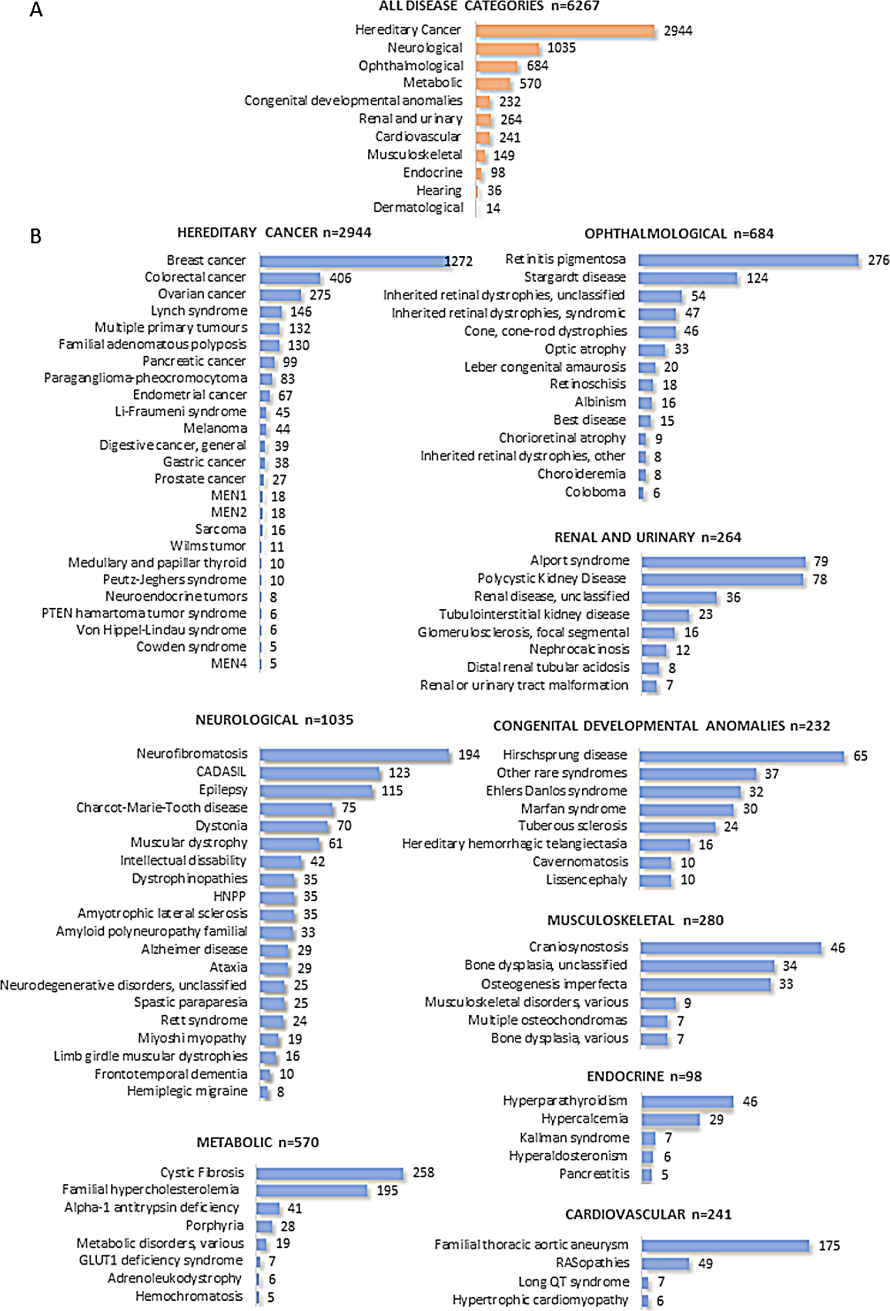
Case distribution per disease category. Distribution of referred genetic tests by disease category based on clinical suspicion and considering 11 different major disease categories. Bars show the size of each case set grouped by disease category (**A**, orange bars) and subcategories (**B**, blue bars). For clarity, case sets with *n* < 5 have been omitted as well as disease categories represented by only one subcategory with *n* ≥ 5 (hearing and dermatological disorders)

A total of 6267 genetically undiagnosed patients with hereditary conditions were sequenced using pRARE (Fig. 1). In addition, 233 individuals were included for reproductive carrier screenings or for the identification of technically challenging variants. Patients were classified into 11 major categories based on their clinical diagnosis and further divided into a variable number of subgroups (Fig. 3). Multisystem conditions were only counted according to the primary referring specialty. Hereditary cancer was the most frequent primary referral diagnosis, followed by neurological and ophthalmological disorders.

### Data quality and pRARE performance evaluation

The three pRARE versions (D1, D2, and D3) showed significant differences in total number of reads (*p* < 0.0001), with D1 showing higher values (Additional file 4 A), primarily due to the inclusion of fewer samples per sequencing run in the initial phase of the pilot program (Additional file 4B). Variations in library preparation procedures (Roche SeqCap Ez vs. Roche KAPA hyperplus kits) and panel sizes also contributed to D3 having the lowest (*p* < 0.0001) mean depth (253x) (Additional file 4 C), although still well above the recommended threshold for germline analyses [29].

The use of KAPA Hyperplus Kit version 3.1 in D3 demonstrated a marked improvement in uniformity (*p* < 0.0001), with a fold 80 base penalty mean of 1.34 (Additional file 4D), approaching the ideal value of 1 which represents perfect uniformity. D3 also outperformed previous designs in reducing duplicate reads (Additional file 4E) and enhancing sensitivity, particularly in terms of panel coverage at a minimum threshold of 20x (Additional file 4 F, *p* < 0.0001). However, the percentage of reads on target did not improve in D3 compared to D1 and D2 (Additional file 4G, *p* < 0.0001), potentially reflecting that the KAPA Hyperplus Kit prioritizes uniformity over specificity.

To identify commonly uncovered regions among the three pRARE versions, coding exons that were covered less than 50% in at least 95% of the samples for each design, were assessed. Incremental improvements across designs were observed, as demonstrated by a reduction in the proportion of uncovered coding regions (Additional file 4 H), and despite the increase in total coding regions. Remarkably, only three genes, *STRC*, *OTOA* and *GFRA2*, retained uncovered exons in all versions of pRARE (Additional file 5).

On the other hand, the resulting EMQN reports gave us the highest score and revealed that each pRARE version progressively increased the values of sensitivity, precision, F-score, uniformity, coverage at 20x and coverage at 30x, while presenting lower than average error rate and percentage of reads off-target (Additional file 6). Additionally, regions with significant homology or pseudogenes initially included in the first version of pRARE were excluded in subsequent versions due to unreliable variant calling.

### Diagnostic yield

**Fig. 4:**
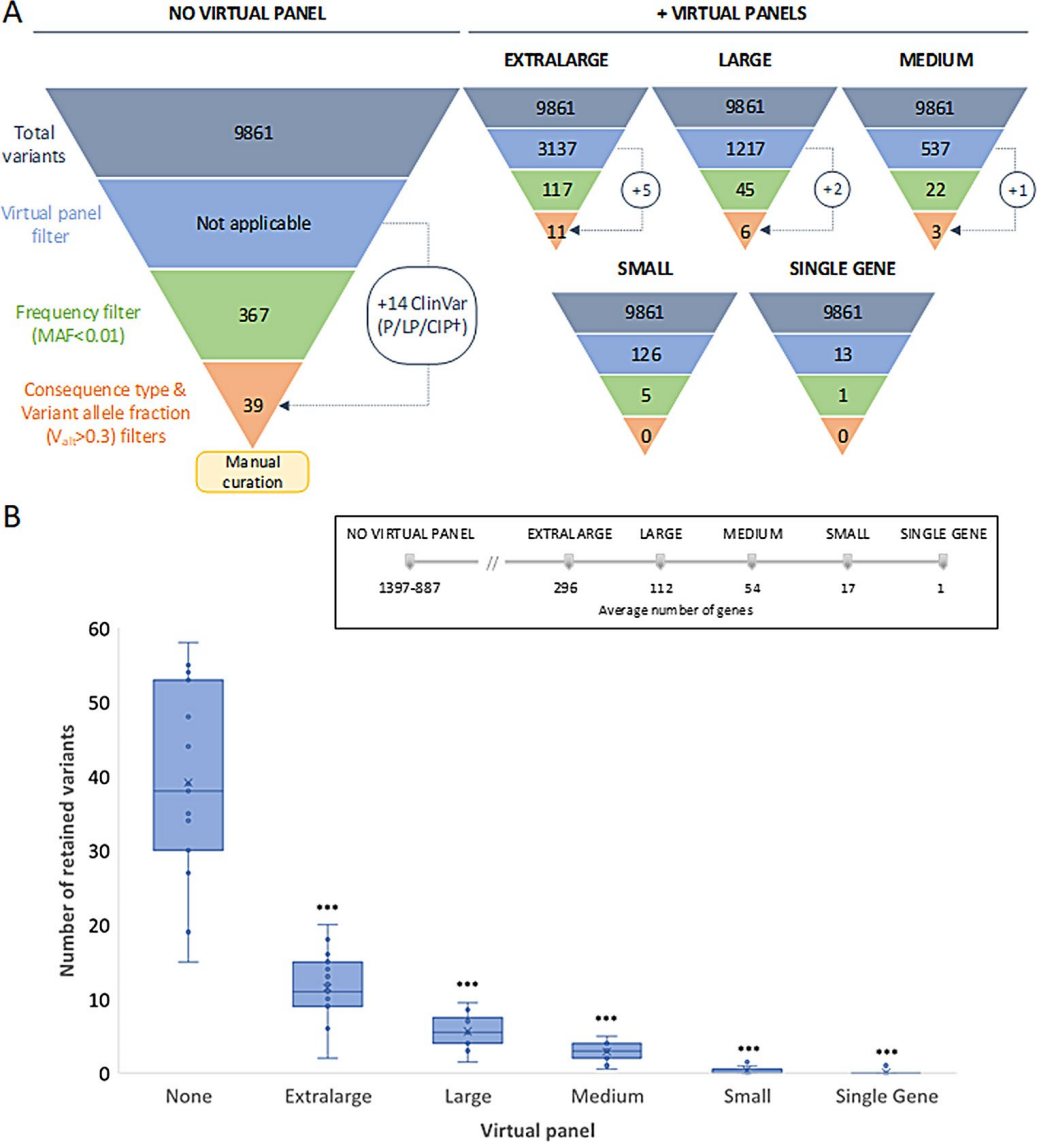
The use of virtual panels is a key step in variant filtering and prioritization. **(A)** Comparison of the mean number of candidate variants in randomly selected patients after applying the different automatic filtering steps with and without the use of virtual panels. **(B)** Boxplot diagram comparing the number of variants retained using our automatic filtering strategy with or without the application of different virtual panels, according to the number of genes included. T-test analysis was performed using the no-panel filtering strategy as the reference group. The application of any-sized virtual panels significantly reduced the number of variants to manually prioritize (p-value < 0,001). Abbreviations: CIP†, conflicting interpretations of pathogenicity with at least one pathogenic or likely pathogenic entry; MAF, minor allele frequency

Sequencing of all genes in the pRARE panel generated an average of 9623 (D1), 8859 (D2), and 11,100 (D3) variants per individual. Only genes associated with the chief complaint were analyzed to facilitate variant analysis and interpretation, as well as to minimize the risk of incidental findings. To this end, a total of 757 (D1), 873 (D2), and 822 (D3) partially overlapping virtual gene panels were generated (Additional file 7), structured into 14 major clinical categories, which were made up of three or more subpanels corresponding to more specific disease groups. The application of virtual panels reduced phenotype-linked candidate variants by 68–99% (Fig. 4). The use of additional filters further reduced the number of variants to be prioritized, ranging from 0 to 11 (Fig. 4A), being these differences statistically significant (Fig. 4B).

**Fig. 5:**
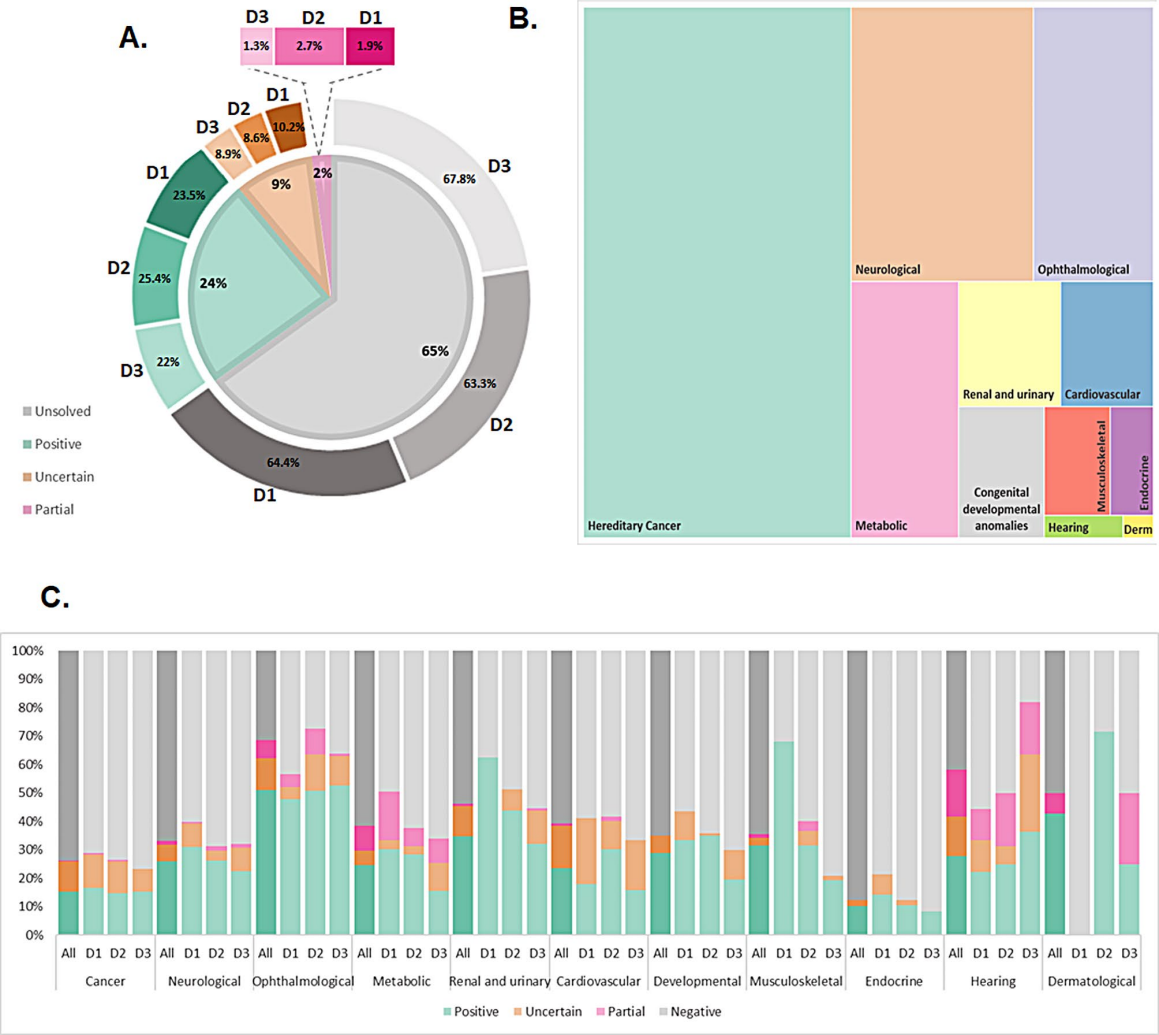
Graphical representation of the main genetic outcomes obtained for this strategy. **(A)** The genetic diagnosis rate for the whole approach and broken down for each of the versions of pRARE. **(B)** Treemap showing the major disease categories, in which each plot is scaled to represent the number of studied cases. Derm: Dermatological. **(C)** Percentage of cases with a full genetic diagnosis (positive), with variants of unknown significance consistent with their phenotypes (uncertain), with a monoallelic likely causative variant in an autosomal recessive gene (partial), and with no candidate variants (unsolved) for each major disease category both overall (“All”; saturated colors) and for the three pRARE versions (“D1”, “D2” and “D3”; blurred colors)

**Fig. 6:**
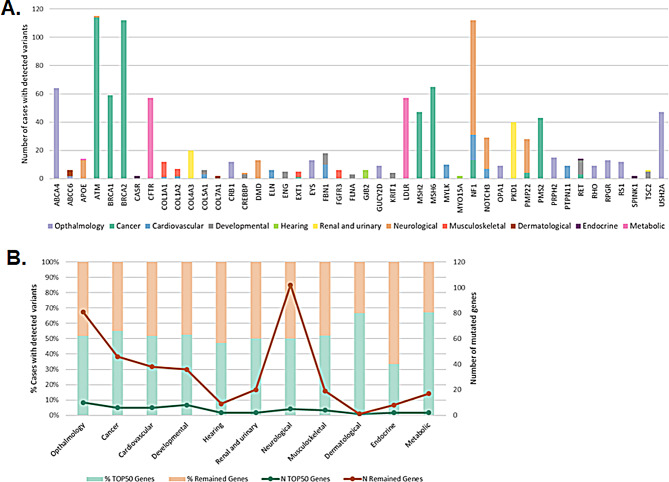
Genetic heterogeneity for each of the main disease categories and prevalence of the mutated genes. “TOP50 Genes” refers to the set of genes that, together, harbor variants that could explain the phenotype for at least 50% of cases with detected variants of each disease category, with the exception of categories “Hearing” and “Endocrine”, in which only two genes were mutated in more than 1 patient resulting in a prevalence < 50% (47% and 33% of cases, respectively). **(A)** Most prevalent mutated genes (TOP50 Genes) in our cohort of patients, showing the recurrence of each of them. The figure also illustrates the prevalence of these genes in different categories, when applicable. **(B)** Depiction of the percentage of cases with variants both in TOP50 Genes and the remained genes for each category (bar chart). The line graphs show the number of genes making up the TOP50 and the number of remained mutated genes per category, illustrating the genetic heterogeneity for each category, which is directly proportional to the distance between the points of both gene groups

After prioritization and laboratory validation, a positive genetic diagnosis was established in 1497 cases out of 6267 index patients (23.9%). This figure may increase to 2061 (32.9%) if uncertain cases ultimately yield positive results (Fig. 5). Detailed data for each disease category and subcategories can be found at Additional file 8. Diagnostic yields varied by main disease categories, ranging from 12% (for endocrine category) to 62% (for ophthalmological category), also dependent on the pRARE version. In total, 353 mutated genes were identified (Additional file 9), showing a high level of genetic heterogeneity. Among these, 46 genes were notably prevalent in our cohort (Fig. 6).

### Comparison between pRARE versions and sample reanalysis

Comparisons among pRARE versions revealed no statistically significant differences in diagnostic yield (Additional file 10). However, 46 cases with genetic results (classified as positives, uncertain, partial and carriers) involved 28 genes that were incorporated during the pRARE updates (D2 and D3) and were consequently absent in the initial version (D1). Additionally, 90 cases, with negative or inconclusive results, were reanalyzed through resequencing using the updated versions of pRARE. As a result, 12% of these patients (*n* = 11) received a definitive genetic diagnosis (Additional file 11). Of these, 6 cases were diagnosed as a result of the inclusion of new causal genes in the panel design, 4 cases were attributed to the refinement of virtual panel content, and 1 case was diagnosed following improvements in the variant prioritization pipeline.

### Clinical impact of the genetic results

**Fig. 7:**
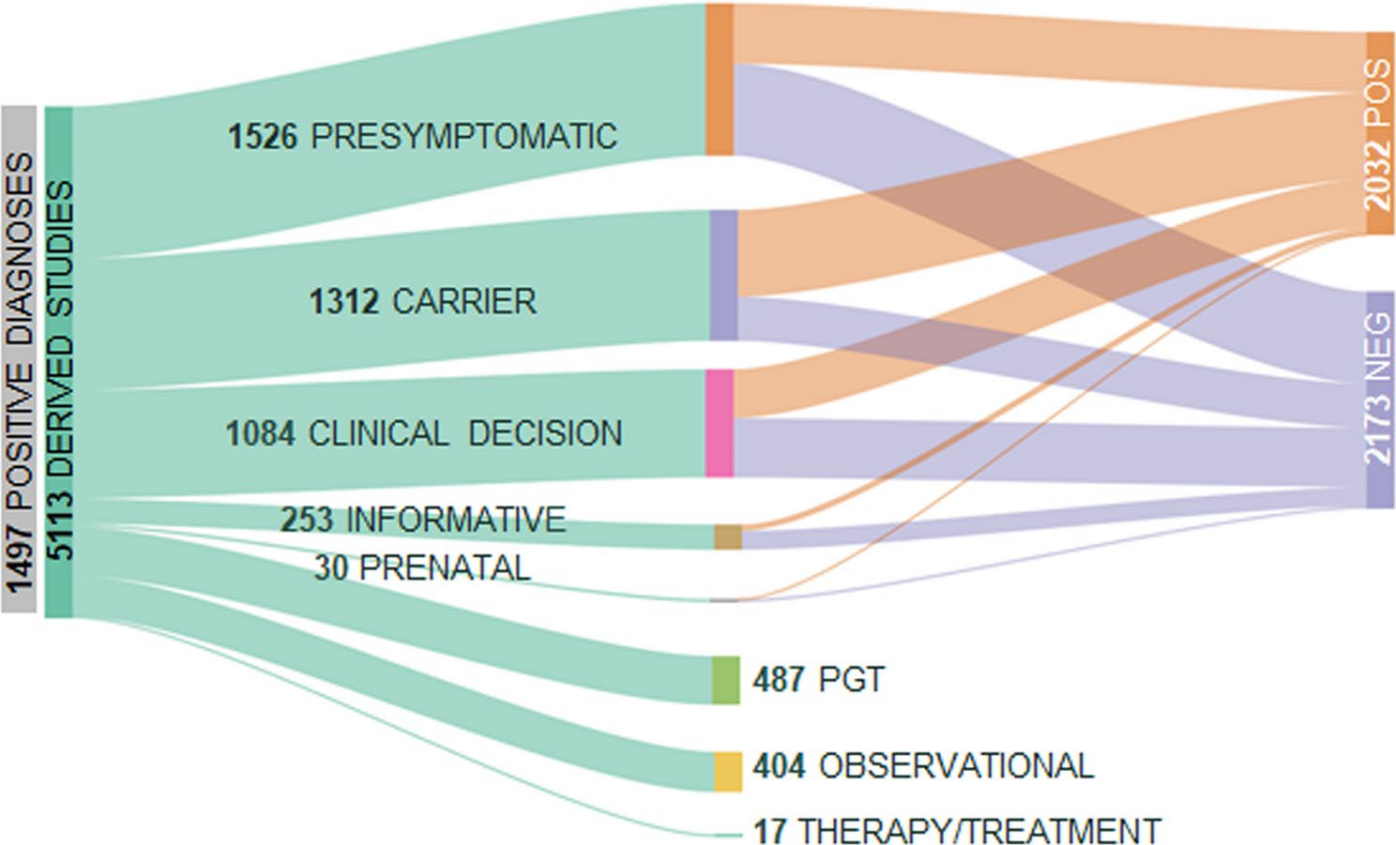
Cases included in observational studies and benefiting from treatments/therapies. Patients with positive diagnostic findings and/or their families were offered to be included in additional studies including carrier screening, presymptomatic, prenatal or preimplantational genetic testing. As a result, a total of 3121 individuals received reproductive genetic counselling, surveillance follow- up or genetic-guided therapeutic decisions. Also, a set of patients were recruited for observational studies based on the molecular results and 17 patients had access to a personalized pharmacological or gene therapy treatment. POS: Positives; NEG: Negatives

Once a genetic diagnosis was established, we used this information to evaluate familial implications, such as identification of at-risk family members, guidance for reproductive decisions or treatment recommendations. This resulted in 5113 non-NGS additional studies (Fig. 7), including 1526 presymtomatic tests, 1312 carrier screenings, 253 informative studies and 30 prenatal diagnoses. Additionally, our results enabled us to recommend 101 preimplantation genetic tests for autosomal recessive monogenic diseases and 272 for autosomal dominant and X-linked disorders. Furthermore, the genetic data guided informed clinical decisions (e.g. screenings, medication options, prophylactic surgery, etc.) for 1084 patients across different disease groups. Also, 404 patients were enrolled in two observational studies and 17 individuals benefited of a treatment/therapy developed for specific genetic variants or genes. Details of this information are presented in Additional file 12.

## Discussion

The diagnostic potential of NGS is evident, yet several challenges must be addressed to ensure its effective implementation into the clinical routine of healthcare systems [30, 31]. Our initiative in Andalusia, which has the potential to integrate clinical and genomic data into an interoperable electronic health record and ePrescribing systems, is an important step towards personalized medicine.

In this approach, we opted for large panels-based sequencing over WES or WGS due to its greater affordability, versatility and sensitivity [32]. Multiple studies have indicated that targeted gene panel sequencing and WES demonstrate comparable diagnostic yields [33], especially in settings where known disease-associated genes are involved [34, 35]. Moreover, panel sequencing has been shown to offer a more rapid and effective screening method than WES for the diagnosis of specific disease groups [6, 33, 36–39], particularly in conditions with high genetic heterogeneity, such as cardiovascular diseases and hereditary cancer, improving clinical outcomes [34, 40].

However, panel-based approaches imply ongoing monitoring of genotype-phenotype associations and regular reanalysis of data to incorporate new genetic discoveries. It is important to note that larger-scale NGS studies are not exempt from this workload, since they also require this knowledge for establishing and updating virtual panels. Currently, this problem has been addressed using widely adopted applications such as Genomics England PanelApp for sharing, accessing and evaluating gene panels for routine genetic diagnostic testing [41]. Consistent with previous research [42], the use of expert-validated gene panels has been essential in our model for maximizing rare disease diagnoses. Regular updates to these panels allowed us to capture emerging genotype-phenotype correlations, which is essential for keeping pace with ongoing discoveries in genomic medicine [43]. This process ensures that genetic data remain clinically relevant as new insights are uncovered in the fields of genomics and precision medicine [44]. This also underscores the importance of multidisciplinary teams in interpreting the clinical significance of newly identified variants, which is essential for the successful implementation of precision medicine within healthcare systems.

Recent studies have highlighted the value of reanalyzing genomic data, showing that periodic re-evaluation can uncover clinically relevant variants that were initially missed or not recognized [31]. This is especially relevant for complex genetic disorders, where new genetic discoveries continuously reshape our understanding of disease mechanisms and often involve intricate genotype-phenotype relationships, requiring a deep understanding of both common and rare genetic variants [45–47]. The refinement of genomic approaches that enable more precise capture of relevant regions and facilitates variants interpretation such as panel-based sequencing, will improve diagnostic accuracy and the development of targeted therapies [48].

Despite the high quality of our data, the use of targeted sequencing limits the detection of certain variant types, such as large structural variants or regions with high homology or pseudogenes, repetitive regions or medium size indels, potentially leading to false negatives [49]. These challenges are consistent with the literature, which emphasizes the need for complementary strategies, such as the integration of multi-omics approaches in complex cases of rare genetic diseases [44, 50, 51]. In fact, multi-omics integration has been proven essential in the study of multifactorial genetic disorders where complex interactions between genetic and other molecular factors are key to understanding pathogenesis and informing treatment options [52, 53]. In this light, the future of clinical sequencing is likely to involve a combination of short- and long-read sequencing, optimizing both diagnostic precision and the detection of different types of challenging variants [54].

Another key aspect of our model is the development of PMM, a bioinformatic tool for the analysis of genomic data in a clinical context. Importantly, although not exploited in this work, PMM also provides functionalities (e.g.: commonly used in silico deleteriousness predictors) to handle larger datasets, such as whole exomes, while maintaining accuracy and improving clinical decision-making. An example of the potential of PMM in research has been highlighted by its application in variant screening of the newly identified retinal disease gene (*THRB*) [55] in unsolved cases of inherited retinal dystrophies, further reinforcing the need for constant updates of virtual panels.

The local processing of data facilitated by PMM ensures compliance with privacy regulations and patient comfort, which is a growing concern with public, commercial platforms [56]. However, reliance on exclusively local data may hinder the implementation and impact of NGS in global clinical practice and research, where cross-border collaboration and genetic data sharing are crucial for accurate diagnosis and treatment [57, 58]. Our solution offers multiple advantages over widely used commercial options in the clinical setting, providing: (i) equity for patients, (ii) scalability and cost reduction for the system, (iii) adaptation to clinical professionals without specific training in genomics, and (iv) the ability to store data locally within the health system. It is noteworthy that these strengths are achieved without compromising the diagnostic rate. In fact, the diagnostic yields achieved (12 − 62%, depending on the phenotype category) equal or improve the results of other genomic approaches for rare inherited conditions [59] and did not significantly differ among the three panel versions. While not statistically significant, other categories such as ophthalmological, hearing, and dermatological also showed improved diagnostic performance, reflecting a positive trend that may have been underestimated due to the heavy dependence of the statistical tests on the sample size. Indeed, the diagnosis of 46 cases (3%) was only achievable due to pRARE updates. This supports the idea that the total number of patients who have benefited from this approach is more illustrative than diagnostic yields.

Common to the three pRARE versions was the high percentage of cases with an uncertain diagnosis. Variants of unknown significance (VUS) represent one of the greatest challenge that geneticists face when applying NGS methods to the diagnosis of rare diseases [60]. In these cases, it is particularly important to develop a monitoring and reanalysis protocol with the aim of reclassifying variants with the most up-to-date scientific knowledge [44, 61]. In fact, our results indicate that data re-analysis could provide a genetic diagnosis in 5% of cases without the need to generate new genomic data. Nevertheless, despite achieving new diagnoses, not all VUS could be resolved, and some gene-disease associations remain unclear. In agreement with other studies, we emphasize the need of continuous reannotation and reclassification to ensure accurate diagnostic outcomes [48]. In addition to these challenges, another key factor in diagnostic accuracy lies in understanding population-specific genetic variations [62], which are critical in addressing disparities in diagnostic rates and outcomes. Generalized diagnostic studies, while valuable, often fail to capture the genetic diversity unique to specific populations [63]. However, regional cohorts can introduce population bias, potentially limiting the generalizability of the findings to broader or more diverse populations, underscoring the need for both types of approaches. For instance, specific variants or genes that are common in other regional groups may be underrepresented, potentially underestimating the global diagnostic utility of the gene panels used. Furthermore, bias in the inclusion criteria according to our services portfolio, may have resulted in an uneven representation of some disease categories, unavoidably skewing the interpretation of diagnostic success in these areas. Nevertheless, while our cohort is regionally focused, it encompasses a highly diverse population, as Andalusia represents around 18% of Spain´s population, reducing the extent of this bias compared to studies conducted in reduced or more genetically isolated cohorts.

The establishment of accessible genomic databases linked to up-to-date clinical information is essential for advancing knowledge and enhancing clinical applications [64]. Based on our current understanding, this is the first corporate information and care management system underway to integrate genomics and medical records in an interoperable public health system. The clinical utility of this strategy extends beyond diagnosis, impacting family screening, reproductive decisions, and therapeutic interventions [65]. Similarly, other studies have emphasized the critical role of genetic testing in guiding clinical decisions, particularly in optimizing therapeutic strategies for complex diseases [66].

## Conclusions

In summary, this strategy has shown promising improvements in the diagnostic rate and response times, contributing to a broader services portfolio within our hospital. The consequent increase in the number of patients benefiting directly or indirectly from genetic studies suggests that this approach is effectively addressing critical clinical needs which could enhance patient outcomes. Furthermore, the self-designed tools have provided a safe, efficient and agile corporate environment, adapted to the daily routine of clinicians and geneticists and facilitating timely clinical decision-making. However, it is essential to acknowledge that while these outcomes are encouraging, the clinical significance of these findings must be interpreted with caution. Future efforts should focus on refining reanalysis protocols and expanding multi-omic approaches to address unsolved cases. Finally, while the integration of clinical and genomic data holds promise for advancing both retrospective and prospective healthcare studies, further transformative innovations are required to fully leverage the benefits of genome sequencing in biomedicine. This includes improving the integration and digitization of information and data management systems, leveraging population-based datasets to generate evidence on precision medicine outcomes, and informing clinical decisions at the point of care.

## Electronic supplementary material

Below is the link to the electronic supplementary material.

Supplementary Material 1

Supplementary Material 2

Supplementary Material 3

Supplementary Material 4

Supplementary Material 5

Supplementary Material 6

Supplementary Material 7

Supplementary Material 8

Supplementary Material 9

Supplementary Material 10

Supplementary Material 11

Supplementary Material 12

## Data Availability

The authors declare that all results obtained are presented in the text, figures and additional files. De-identified, aggregated data can be obtained upon reasonable request from the corresponding authors (gantinolo@us.es; salud.borrego.sspa@juntadeandalucia.es).

## Abbreviations

NGS: NextGeneration Sequencing
PMM: Personalized Medicine Module
pRARE: Personalized Rare Disease Exome
gDNA: genomic DNA
CNVs: Copy Number Variations
MLPA: Multiplex Ligation-dependent Probe Amplification
qPCR: quantitative PCR
ACMG: American College of Medical Genetics and Genomics
AMP: Association for Molecular pathology
WES: Whole Exome Sequencing
WGS: Whole Genome Sequencing
RTHβ: Resistance to Thyroid Hormone beta
VUS: Variants of Unknown Significance
