# Supplementary material for: A genomic strategy for precision medicine in rare diseases: integrating customized algorithms into clinical practice
Source: J Transl Med. 2025 Jan 20;23:86. doi: 10.1186/s12967-025-06069-2 (PMC11748347; doi:10.1186/s12967-025-06069-2)
Supplement: Supplementary file 1 — Supplementary Material 1 [file 12967_2025_6069_MOESM1_ESM.docx]

A Genomic Strategy for Precision Medicine in Rare Diseases: Integrating Customized Algorithms into Clinical Practice

*Cristina Méndez-Vidal^1,2^*^†^*, Nereida Bravo-Gil^1,2^*^†^*, Javier Pérez-Florido^1,2,3^, Irene Marcos-Luque^2,4^, Raquel M. Fernández^2,4^, José Luis Fernández-Rueda^3^, María González-del Pozo^1,2^, Marta Martín-Sánchez^1,2^, Elena Fernández-Suárez^1,2^, Marcela Mena^1,2^, Rosario Carmona^1,2,3^, Joaquín Dopazo^1,2,3^, Salud Borrego^2,4*^, Guillermo Antiñolo^2,4*^*

^1^ Institute of Biomedicine of Seville, IBiS/University Hospital Virgen del Rocio/ CSIC/University of Seville. Seville. Spain.

^2^ Centre for Biomedical Network Research on Rare Diseases (CIBERER), Seville, Spain.

^3^ Platform of Computational Medicine. Fundación Progreso y Salud (FPS). CDCA, University Hospital Virgen del Rocio. Seville. Spain.

^4^ Department of Maternofetal Medicine, Genetics and Reproduction. Institute of Biomedicine of Seville, IBiS/University Hospital Virgen del Rocio/CSIC/University of Seville. Seville. Spain.

† These authors contributed equally to this work and they must be considered as joint first authors.

* Corresponding author.

**ADDITIONAL FILE 1**

# SUPPLEMENTARY METHODS

## DNA extraction and quality assessment

Genomic DNA was extracted from peripheral blood samples using automatic DNA extraction platforms (Chemagic™ 360, Perkin Elmer, MA, USA, or MagNA Pure LC system, Roche, IN, USA), following the manufacturer’s instructions. DNA purity (OD260/280 and OD230/260 ratios) was measured using a NanoDrop ND-1000 spectrophotometer (Thermo Fisher Scientific, Wilmington, DE, USA). DNA quality and integrity were assessed by electrophoresis on a 1% agarose gel and DNA quantification was performed using fluorometric methods (Quant-iT PicoGreen, Invitrogen, Carlsbad, CA, USA or Qubit™ 1X dsDNA HS, Thermo Fisher Scientific, Waltham, MA, USA).

## Design and development of a personalized Rare Diseases Exome (pRARE)

A detailed view of the three targeting sequencing panels, including the total region covered or the underlying technology is shown in Supplementary Table 1. Clinically relevant genes for diagnosis were selected following current guidelines for the diseases, GeneReviews, the Online Mendelian Inheritance in Man (OMIM), Orphanet, and a review of the literature. An isoform for each transcript was selected using the information from HGNC, UCSC and NCBI RefSeq databases and manual literature curation (Supplementary Table 1B). Regions with significant homology or pseudogenes (e.g. *SMN1*, *SMN2*, *PMS2*, and *CYP21A2*) were included in the initial version of pRARE (D1) to assess the detection of challenging variants using our NGS-based workflow. Probes were designed against the human NCBI GRCh37/hg19 reference genome assembly. The UCSC genome browser was used to generate a list of genomic coordinates for the target regions which was uploaded to the NimbleDesign (D1 and D2) or HyperDesign (D3) applications (Roche, Madison, WI, USA) for probes design.

The diagnostic yield obtained for each of the main categories was statistically compared among the three versions of pRARE, by using the IBM SPSS Statistics for Windows Version 29.0 (IBM Corp., Armonk, NY, USA) and the R statistical software. Descriptive statistics were used, representing the absolute and relative values of the qualitative variables as well as measures of central tendency and variability for the quantitative variables. In terms of inferential statistics, the assumption of normality of the variables was verified. For this study, we applied generalized mixed models to compare D1 vs D2 and D2 vs D3, except for categories “hearing” and “dermatological”, in which the sample size was insufficient. The calculations were performed with the maximum diagnostic rates of each category, including both positive and uncertain cases. The obtained results were considered statistically significant for two-sided p-values adjusted for Bonferroni correction < 0.05.

## Library preparation and sequencing

One microgram of genomic DNA was used for library preparation using the SeqCap EZ Library SR version 5.1 for D1 and D2, or KAPA Hyperplus Kit version 3.1 for D3 (Roche, Indianapolis, IN, USA) according to the manufacturer’s protocol with minor modifications. For D1 and D2, DNA was sheared by mechanical fragmentation using a Covaris S220 instrument (Covaris, Woburn, MA, USA) to obtain an average fragment size of 180–220 bp. For D3, the KAPA Hyper Plus kit for enzymatic fragmentation was used. Fragments were end-repaired, A-tailed, and ligated to specific adapters. After library normalization, samples were dual indexed (KAPA Dual-Indexed Adapter kit), pooled (up to 60 libraries for D1 and D2 or 50, for D3) and 5 μg of DNA were hybridized to biotinylated probes for 16–20 h at 47 °C. The biotinylated DNA probes were recovered and purified using streptavidin-conjugated magnetic beads and PCR amplified. Quantification of libraries was made using Agilent 2100 Bioanalyzer (Agilent Technologies, CA, USA) and fluorimetric techniques. Captured libraries were sequenced in multiplexed sequencing runs on the Illumina NextSeq500 sequencer (Illumina, San Diego, CA, USA) as 150 bp paired-end reads using a NextSeq HighOutput v2.5 (300 cycles) reagent kit.

## Primary and secondary analysis of NGS data

Raw sequences (FASTQ files) corresponding to each sample were generated using Illumina *bcl2fastq* conversion software for the primary analysis. A pipeline for processing the FASTQ files based on GATK best practices [1] was applied for the discovery of SNVs and small indels (<50bp). For each sample, FASTQC [2] was used to assess the quality of raw data, and *fastp* [3] was run for quality pre-processing so that clean data was provided for downstream analysis. Then, filtered sequence reads were aligned to the reference human genome build hs37d5 (hg19) by using the BWA alignment tool [4]. Mapped reads (BAM files) were then sorted using *samtools* [5] and duplicate reads were marked to mitigate biases introduced by data generation steps such as PCR amplification through *Picard tools* [6]. BAM files were analyzed in terms of QC using in-house scripts for coverage analysis and the *ngsCAT* tool [7]. Then, a recalibration of base quality score (BQSR) procedure from GATK was run to detect and correct patterns of systematic errors in the base quality scores. After BQSR, variants (SNVs and small indels) were identified using GATK *HaplotypeCaller* and, to reduce putative false positives, a variant filtering step in run based on GATK’s hard filters. The result of this pipeline was a VCF file for each sequenced sample.

On the other hand, CNVs were identified employing the coverage command of BEDtools, which generates a CSV file. In this method, the number of reads for each chromosomal interval of the bed file was normalized using the average number of reads generated per sample and compared among the other samples from the same sequencing run. As a result, we obtained ratios indicating the gene dose of a patient for each region and a z-score that represents variability among samples calculated based on the standard deviations from the mean.

The processed genomic data files and raw sequencing data were stored in secured infrastructures with long-term capabilities at the Computational Medicine Platform within the University Hospital Virgen del Rocío.

## Tertiary analysis of identified variants

Coding non-synonymous and splicing variants (8bp intronic and 2bp exonic), with no minor allele frequency (MAF) or below 0.01 in 1000GP, the Exome Aggregation Consortium (ExAC), the Genome Aggregation Database (GnomAD), Exome Variant Server (EVS) and the Collaborative Spanish Variant Server (CSVS), were selected for further analysis. Variants with an internal recurrence greater than two were also discarded when the matching inheritance pattern was dominant as long as there was no phenotypic concordance. Additionally, variants with at least one pathogenic or likely pathogenic ClinVar entry were also considered regardless of their location, frequency and recurrence. To discard genetic artifacts, variants with an allele fraction less than 30% were also filtered out. The remaining variants were also prioritized based on their zygosity and the mode of inheritance associated with the mutated gene according to OMIM and the literature. Besides the PMM-integrated database ClinVar (<https://www.ncbi.nlm.nih.gov/clinvar/>), the clinical significance of known variants was further assessed using Leiden Open Variation Database version 3.0 (LOVD; https://www.lovd.nl/) and Human Gene Mutation Database (HGMD; http://www.hgmd.cf.ac.uk). Variants classified as benign or likely benign without conflicting interpretations in these databases were discarded. In addition, with the exception of variants previously reported as pathogenic, hypomorphic alleles, low-penetrance variants, and variants showing variable expressivity, the number of homozygotes, heterozygotes, and hemizygotes in gnomAD was checked. Variants detected in homozygous/hemizygous state or heterozygous in control individuals were ignored for recessive and dominant cases, respectively. Regarding CNVs, the prioritization was based on ratios <0.65 for deletions and >1.35 for insertions/duplications and on the highest absolute z-score values. All identified CNVs were inspected with Integrative Genomics Viewer (IGV) and checked in the Database of Genomic Variants (DGV), HGMD and gnomAD.

# BIBLIOGRAPHY

1. Van der Auwera GA, Carneiro MO, Hartl C, Poplin R, Del Angel G, Levy-Moonshine A, et al. From FastQ data to high confidence variant calls: the Genome Analysis Toolkit best practices pipeline. Curr Protoc Bioinformatics. 2013;43(1110):11 0 1- 0 33.

2. Andrews S. FastQC: a quality control tool for high throughput sequence data. Available online at: <http://www.bioinformatics.babraham.ac.uk/projects/fastqc>. 2010.

3. Chen S, Zhou Y, Chen Y, Gu J. fastp: an ultra-fast all-in-one FASTQ preprocessor. Bioinformatics. 2018;34(17):i884-i90.

4. Li H, Durbin R. Fast and accurate short read alignment with Burrows-Wheeler transform. Bioinformatics. 2009;25(14):1754-60.

5. Li H, Handsaker B, Wysoker A, Fennell T, Ruan J, Homer N, et al. The Sequence Alignment/Map format and SAMtools. Bioinformatics. 2009;25(16):2078-9.

6. Ebbert MT, Wadsworth ME, Staley LA, Hoyt KL, Pickett B, Miller J, et al. Evaluating the necessity of PCR duplicate removal from next-generation sequencing data and a comparison of approaches. BMC Bioinformatics. 2016;17 Suppl 7(Suppl 7):239.

7. Lopez-Domingo FJ, Florido JP, Rueda A, Dopazo J, Santoyo-Lopez J. ngsCAT: a tool to assess the efficiency of targeted enrichment sequencing. Bioinformatics. 2014;30(12):1767-8.
