# Supplementary material for: A genomic strategy for precision medicine in rare diseases: integrating customized algorithms into clinical practice
Source: J Transl Med. 2025 Jan 20;23:86. doi: 10.1186/s12967-025-06069-2 (PMC11748347; doi:10.1186/s12967-025-06069-2)
Supplement: Supplementary file 4 — Supplementary Material 4 [file 12967_2025_6069_MOESM4_ESM.pdf]

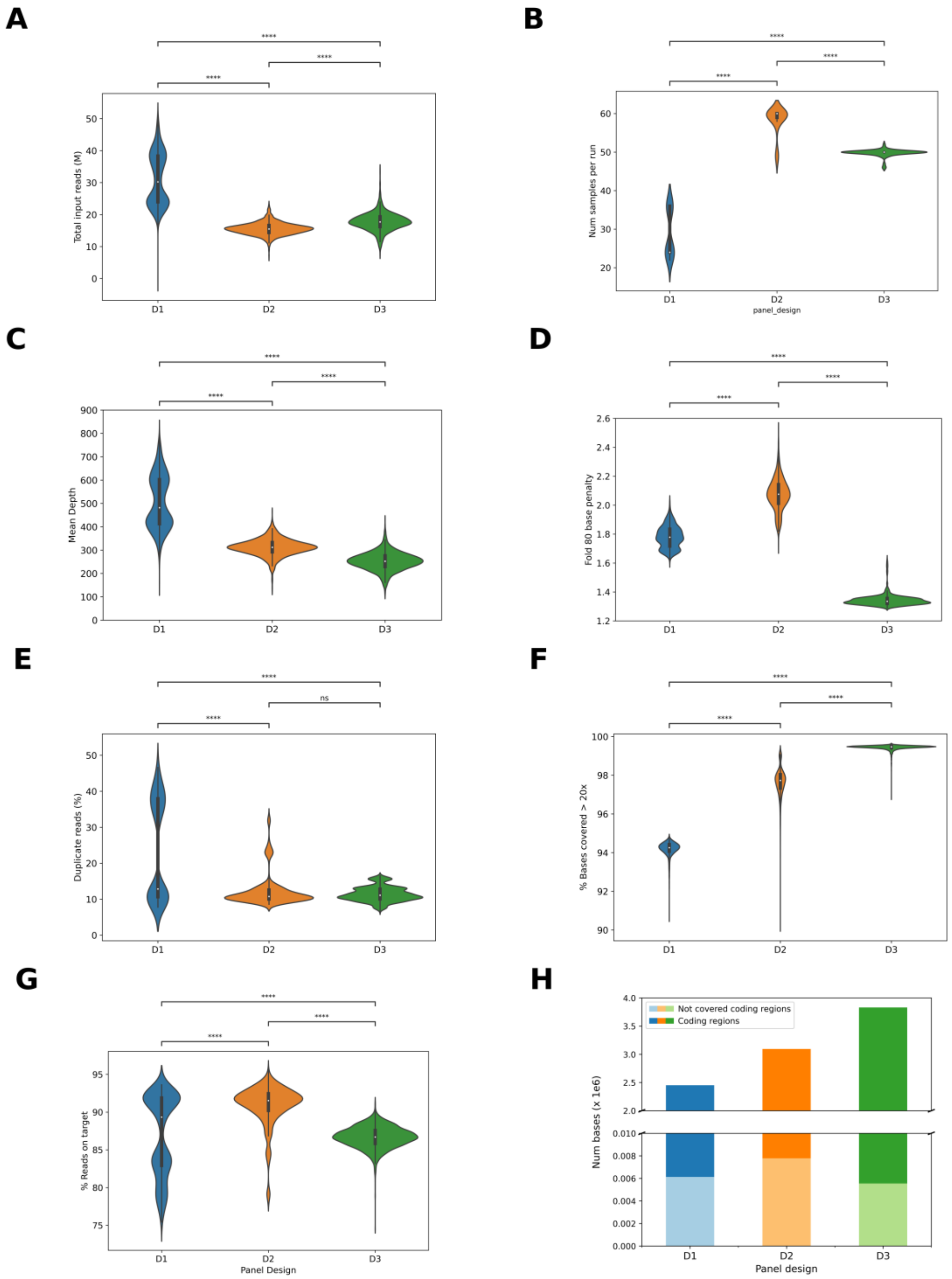

Additional File 4. Performance metrics for D1, D2 and D3. A-G. Violin plots presenting data comparisons between D1, D2 and D3 for **A**. Total input reads (in millions; M) **B**. Number of samples per sequencing run **C.** Mean Depth **D.** Fold 80 base penalty **E.** Duplicate reads **F.** % Bases covered > 20x **G.** % Reads on target. **** $p < 0.0001$ and ns = not significant, according to comparisons between two groups using the Wilcoxon test. A significance threshold of $p < 0.05$ was set to determine whether results are significantly different **H.** Bar plots presenting coding regions that were covered less than 50% in at least 95% of the samples for each pRARE version within the corresponding cohort.
