# Supplementary material for: A genomic strategy for precision medicine in rare diseases: integrating customized algorithms into clinical practice
Source: J Transl Med. 2025 Jan 20;23:86. doi: 10.1186/s12967-025-06069-2 (PMC11748347; doi:10.1186/s12967-025-06069-2)
Supplement: Supplementary file 11 — Supplementary Material 11 [file 12967_2025_6069_MOESM11_ESM.pdf]

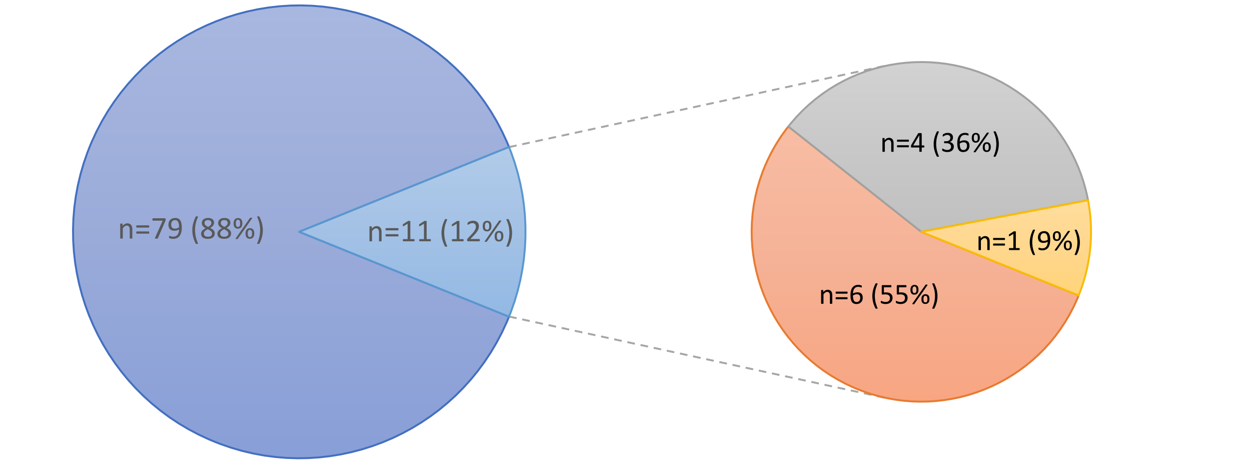

Additional File 11. Diagnostic yield of patients who underwent resequencing using updated versions of pRARE and analysis of how different improvements in panel design had contributed to the genetic diagnosis.
